# *Alpiniae oxyphyllae* fructus possesses neuroprotective effects on H_2_O_2_ stimulated PC12 cells via regulation of the PI3K/Akt signaling Pathway

**DOI:** 10.3389/fphar.2022.966348

**Published:** 2022-08-25

**Authors:** Ruolan Li, Lingyu Wang, Qing Zhang, Huxinyue Duan, Die Qian, Fei Yang, Jun Xia

**Affiliations:** ^1^ School of Pharmacy, School of Basic Medicine, Chengdu University of Traditional Chinese Medicine, Chengdu, China; ^2^ Hospital of Chengdu University of Traditional Chinese Medicine, Chengdu, China

**Keywords:** alzheimer’s disease, Alpiniae oxyphyllae fructus, network analysis, apoptosis, PI3K, akt

## Abstract

**Backgroud:** Alzheimer’s disease (AD) is a typical neurodegenerative disease, which occurs in the elderly population. *Alpiniae oxyphyllae* Fructus (AOF) is a traditional Chinese medicine that has potential therapeutic effect on AD, but the mechanism behind it is unclear.

**Methods:** Firstly, the main chemical components of AOF were identified by LC-MS, while the main active ingredients and targets were screened by TCMSP database. At the same time, AD-related target proteins were obtained using Genecards and OMIM databases. PPI was constructed by cross-linking AOF and AD targets, and GO enrichment analysis and KEGG pathway enrichment analysis were performed to identify the relevant biological processes and signaling pathways. Finally, based on the H_2_O_2_-stimulated PC12 cell, flow cytometry, WB and immunofluorescence experiments were performed to verify the protective effect of AOF on AD.

**Results:** We identified 38 active ingredients with 662 non-repetitive targets in AOF, of which 49 were potential therapeutic AD targets of AOF. According to the GO and KEGG analysis, these potential targets are mainly related to oxidative stress and apoptosis. The role of AOF in the treatment of AD is mainly related to the PI3K/AKT signaling pathway. Protocatechuic acid and nootkatone might be the main active ingredients of AOF. In subsequent experiments, the results of CCK-8 showed that AOF mitigated PC12 cell damage induced by H_2_O_2_. Kits, flow cytometry, and laser confocal microscopy indicated that AOF could decrease ROS and increase the activity of superoxide dismutase (SOD), catalase (CAT) and glutathione peroxidase (GSH-Px), while AOF could also increase mitochondrial membrane potential (MMP), thereby inhibiting apoptosis. Finally, immunofluorescence and WB results showed that AOF inhibited the expression of BAX and caspase-3 in PC12 cells, and promoted the expression of Bcl-2. At the same time, the phosphorylation levels of PI3K and Akt proteins were also significantly increased.

**Conclusion:** This study suggests that AOF had the potential to treat AD by suppressing apoptosis induced by oxidative stress via the PI3K/Akt pathway.

## Introduction

Alzheimer’s disease (AD) is a common neurodegenerative disease and a leading cause of senile dementia (Nixon, 2007; Kern et al., 2018). According to the latest data from the World Alzheimer Report, about 10% of people above 65 years old worldwide are living with AD, and the number is increasing year by year (Alzheimer’s & Dementia, 2022; Gustavsson et al., 2022). The early clinical manifestations of AD patients are memory loss and cognitive dysfunction. Without timely intervention, patients will also have executive dysfunction and even loss of self-care ability (McKhann et al., 2011; Lane et al., 2018). Globally, there have been many studies regarding finding novel drug for AD, but up to now, there is still no drug that can radically cure AD (Huang et al., 2018). Although the US Food and Drug Administration (FDA) has recently approved Aduhelm (aducanumab), a monoclonal antibody targeting amyloid beta (Aβ), its therapeutic efficacy is controversial (Zhang et al., 2022). In addition, many preclinical results on AD have not been translated into clinical application, which also brings great challenges to the treatment of AD (Huang et al., 2022). According to relevant reports, in the year of 2020, the total cost of AD treatment in the United States is about $305 billion, which brings great economic burden to patients and society (Zhang et al., 2022). Therefore, it is urgent to finding more novel drugs which are safe and effective to treat AD.

It is known that Chinese medicine has a long history for medical application in China. At the same time, due to the natural advantages of Chinese medicine with multiple components and multiple targets, its traditional application in modern medicine has been continuously expanded (Li et al., 2022). *Alpiniae oxyphyllae* fructus (AOF), which also called ‘Yizhi’, is the dry ripe fruit of *Alpinia oxyphylla* Miq. has a long history of application in traditional clinical Chinese medicine (Wang et al., 2022). In traditional Oriental medicine, AOF is widely used in vomiting, diarrhea, frequent urination, spermatorrhea and so on (Wang et al., 2022). As research on AOF continues, there are more chemical components identified from AOF, which contains essential oils, sesquiterpenes (e.g., Nootkatone), flavones (e.g., Tectochrysin, Izalpinin, Chrysin, and Kaempferide), diarylheptanoids (e.g., TYakuchinone A and B and Oxyphyllacinol), glycosides, steroids and so on (Chen et al., 2014; Zhang et al., 2015). In addition, more effects of AOF have been reported, such as, anti-cancer, anti-inflammatory, analgesic, anti-allergic, insecticidal, anti-parasitic, anti-microbial and anti-oxidant properties (Yu et al., 2003; Zhang et al., 2011; Chang et al., 2017; Qi et al., 2019). Similarly, AOF was surprisingly found to have anti-AD effects, such as Shenqi Yizhi Decoction and Yizhi Decoction (Liu and Hong, 2017; Wang et al., 2022). However, up to now, there is a lack of research on the effective chemical components of AOF for the treatment of AD and their related mechanisms. In Xu’s experiment, although network pharmacology was used to preliminarily explore the therapeutic effect of AOF, the efficacy and mechanism were not verified through experiments (Xu et al., 2020). In addition, in other experiments, researchers paid more attention to the single mechanism of AOF in treating AD, and did not conduct an overall exploration of the therapeutic effect of AOF (Wang et al., 2018). Therefore, it is of profound significance for the development of AOF to discover the effective compounds and relevant mechanisms in AOF.

Due to the complex chemical composition and synergistic effects of Chinese medicines, a pharmacological experiment that deals only with a single compound or extract are unable to systematically study their pharmacological mechanisms, while the network pharmacology has successfully solved this challenge (Ma et al., 2015; Shao et al., 2022). As an emerging technology, network pharmacology combines the advantages of various disciplines such as systems biology, multidirectional pharmacology, network analysis, and computational biology (Hopkins, 2008; Khan and Lee, 2022). Based on the interactions between drugs, components, targets and diseases, network analysis is used to predict the potential active ingredients and mechanisms, and to construct visual interaction networks, thus revealing the complex mechanisms embedded in Chinese medicine (Li and Zhang, 2013). Therefore, in this study, a network pharmacology approach will be used to screen the active ingredients in AOF with therapeutic effects on AD, predict the potential therapeutic targets and related mechanism pathways, and provide a reference for the development of AOF for the treatment of AD.

### Experiment

#### Chemicals, reagents, and materials

AOF used in the present study was purchased from Tong Ren Tang Co., Ltd (Beingjing, China). Fetal bovine serum (FBS), RPMI-1640 culture medium, phosphate buffer saline (PBS) and 0.25% trypsin-EDTA (1x) were purchased from Gibco Co. (Grand Island, NY, United States). H_2_O_2_ was obtained from Sigma-Aldrich Co. Ltd (Darmstadt, Germany). Dimethyl sulfoxide (DMSO), BCA protein assay reagents and cell counting kit-8 (CCK-8) were purchased from Boster Biol. Tech (Wuhan, China). The assay kits for LDH, CAT and GSH-PX were purchased from the Nanjing Jiancheng Bioengineering Institute (Nanjing, China). The assay kits for DCFH-DA and 5,5′,6,6′-Tetrachloro-1,1′,3,3′-tetraethyl-imidacarbocyanine iodide (JC-1) was obtained from the US Everbright Inc (Suzhou, China). Annexin V-FITC/PI assay kit was purchased from 4A Biotech., Ltd (Beijing, China). All other reagents used in the experiments were of analytical grade. Primary antibodies for PI3K, phosphorylation (p)-PI3K, AKT, p-AKT and horseradish peroxidase-(HPR-) conjugated secondary antibody were obtained from the ABclonal Biotechnol. Co. (Wuhan, China).

### Preparation of AOF and HPLC-MS analysis

Firstly, 10 g of AOF was crushed and soaked for 4 h using 75% ethanol-H_2_O, followed by three times extracts using 100 ml 75% ethanol-H_2_O for 4 h each time. The solution was filtered under reduced pressure to obtain the crude extract of AOF, and subsequently concentrated using a vacuum rotary evaporator at 40°C. Finally, the extract was freeze-dried in a lyophilizer to obtain AOF powder, which was used for subsequent experiments (23% yield). The crude extract of AOF was filtered through a 0.22 μm filter membrane and then directly used for LC-MS detection.

The sample was separated on a UHPLC BEH C18 column (2.1 × 100 mm, 1.7 μm; Waters, Milford, MA, United States) eluted with a mixture of water (A) and acetonitrile (B). The flow rate was 0.3 ml/min, and the injection volume was 3 μL. The gradient was 0–2 min, maintained at 5% B; 2–27 min, increased linearly to 100% B; 27–29 min, maintained at 100% B; 29–30 min, decreased to 5% B, and maintained at 5% B for 3 min. The column temperature was set at 35°C.

Mass detection was undertaken on a Thermo Q-Exactive Orbitrap Mass Spectrometer (Thermo Fisher) equipped with an electrospray ionization source in positive and negative ion modes. Mass conditions were set as follows: capillary temperature, 150°C; spray voltage, 2.5 kV for positive and 2.0 kV for negative ion modes; collision energy, 15–35 eV. The full-scan mass spectrum was recorded in m/z 50–1,200 at seven spectra/s. MS/MS experiments were set as data-dependent scans. All data acquisitions were controlled by Thermo Xcalibur 4.0.27.

The chemical composition of AOF was analyzed by LC-MS system, and the relative molecular mass of primary mass spectrometry was obtained. Then, combined with the secondary fragment ions, the molecular formula was fitted and compared with the database, and the chemical composition and structure of the AOF were further deduced according to the retention time, references or databases.

All results were processed using MassLynx™ (V4.2)

### Network analysis

#### Targets prediction of the identified constituents in AOF

Compounds were drawn using ChemDraw professional 15.0, and the files were saved in Mol. format and uploaded to the Traditional Chinese Medicine Systems Pharmacology database and Analysis Platform (TCMSP, https://old.tcmsp-e.com/index.php) and Swiss Target Prediction (http://www.swisstargetprediction.ch/) to predict the potential targets of the active compounds in AOF (Ru et al., 2014).

### Protein-protein interaction

The key word “Alzheimer’s disease” was input into the Online Mendelian Inheritance in Man database (OMIM) and the Genecards database (http://www.genecards.org) to obtain therapeutic targets related to AD. The target name was converted into gene name through UniProt database (http://www.uniprot.org/) **(** Tang et al., 2015 **)**. At finally, the intersection of the screened potential targets of AOF and the therapeutic targets of AD was analyzed in STRING database (https://sting-db.org/). The PPI analysis results were imported into Cytoscape software (ver.3.7.1), while CytohHubba was used to analyze its hub genes. The parameters “Betweenness Centrality,” “Closeness Centrality,” and “Degree” were calculated to assess the topological importance of the nodes in the PPI network.

### Drug-molecular-target-disease network construction

The compounds in AOF and targets were uploaded into Cytoscape software (ver.3.7.1) to construct the Drug-Molecular-Target-Disease Network (DMTD) diagram. In this network diagram, edges represented the relationships between them. Finally, the importance of each node in the network was evaluated by calculating the degree, average shortest path length and closeness centrality in DMTD.

### Analyses of pathway enrichment

The clusterprofiler software package was downloaded in R language software to analyze the gene ontology (GO) term function and the Kyoto Encyclopedia of genes and genomes (KEGG) pathway enrichment, so as to obtain the function and pathway of the collected proteins **(** Duan et al., 2022 **)**. In this study, the criterion of statistically significant was *p* < 0.05, and only the top 10 were shown in the results.

### Experimental validation

#### Cell culture and treatment

The PC12 cells were purchased from Wuhan Pu-nuo-sai Life Technology Co. Ltd (Wuhan, China) and used throughout the study. PC12 cells were cultured in RPMI-1640 medium containing 10% FBS (v/v), penicillin (100 units/mL) and streptomycin (100 μg/ml) at 37°C in a humidified atmosphere of 5% CO_2_. The medium was changed every other day and the cells were passaged when they reached the exponential growth phase.

PC12 cells were pretreated with different concentrations of AOF, protocatechuic acid and nootkatone for 2 h, and then incubated with 90 μM H_2_O_2_ for another 4 h **(** Li et al., 2020 **)**. Moreover, the AOF was replaced by the same amount of 1,640 medium and then stimulated with 90 μM H_2_O_2_ in model group.

### Determination of cell viability

In this study, Cell Counting Kit-8 (CCK-8) was used to test cell activity. In order to detect the cytotoxicity of AOF, cells were treated with different concentration of AOF (10, 20, 40, 60, 80 and 100 μg/ml) after 24 h of growth. In addition, to verify the protective effect of AOF on PC12 cells under H_2_O_2_ stimulation in subsequent experiments, we also pretreated cells with different concentrations of AOF (10, 20, 40, 60, 80 and 100 μg/ml) for 2h, and then stimulated PC12 cells with 90 μM H_2_O_2_ for 4 h.

### Acridine orange/ethidium bromide (AO/EB) staining

After PC12 cells were treated as described above, AO/EB staining is performed according to the manufacturer’s instructions (Yuanye Biological Technology, Shanghai, China). Briefly, AO and EB staining solution were mixed in equal proportions and then co-incubated with treated PC12 cells in the dark for 15 min, followed by observation of the morphology under a laser confocal microscope (Leica, SP8 SR, Wetzlar, Germany).

### Apoptosis assay by flow cytometer

For this part of the experiment, PC12 cells were inoculated in 6-well plates at a density of 1×10^5^/well and then treated as described above. Subsequently, the cells were collected in a centrifugal tube using EDTA-free trypsin. After washing with PBS to resuspend the cells at 4 °C, the cells were diluted using AnnexinV binding buffer. Finally, 2.5 μL AnnexinV-FITC and 2.5 μL PI staining solution were added. PC12 cells were detected using a FACSCanto Ⅱ Flow cytometer (BD Company, New York, NY, United States ) immediately after the mixture was evenly mixed.

### Assessment of mitochondrial membrane potential

A decrease in mitochondrial membrane potential (MMP, △*Ψ*m) is not only an important marker of mitochondrial dysfunction, but also an early event of apoptosis. In this experiment, PC12 cells were labeled with JC-1, a cationic fluorescent dye localized exclusively in mitochondria. Therefore, after cells were treated in accordance with the above methods, JC-1 was used to co-incubate with cells in the dark for 15min. In addition, the fluorescence intensity was measured under laser confocal microscope after the cells were cleaned twice with PBS.

### Detection of intracellular ROS accumulation

In this part of the experiment, DCFH-DA was used to detect the intracellular ROS content. Briefly, PC12 cells were treated according to the above steps and incubated with 10 μM DCFH-DA for 30 min in the dark and then collected into flow tubes. FACSCanto Ⅱ Flow cytometer (BD Company, New York, NY, United States ) was used to detect the fluorescence intensity of DCF and analyze the intracellular ROS.

Determination of MDA, SOD, GSH-Px and CAT.

In this parts, commercial assay kits were used to assay MDA, SOD, GSH-Px and CAT according to the manufacturer’s instructions. In a nutshell, the cells were incubated in 6-well plates and given different concentrations of AOF and H_2_O_2_ as described above. Immediately afterwards, the washed PC12 cells were lysed using lysis buffer and the total protein was collected. Finally, MDA, SOD GSH-PX and CAT were detected according to relevant instructions.

### Immunofluorescence assay

The PC12 cells were seeded in laser confocal plates and treated with AOF and H_2_O_2_ as described above. After the cells were cleaned by PBS for 3 times, the cells were incubated with paraformaldehyde for 20min, and then infiltrated with 0.3% Triton for 30min after being cleaned again. Subsequently, PC12 cells were incubated with 10% serum for 1h, and incubated with diluted primary antibodies PI3K, p-PI3K, Akt, p-Akt, Bcl-2, Bax and caspase3 (1:200) overnight, respectively, and treated with corresponding fluorescent secondary antibodies (1:150) for 2 h. Finally, after the cells were cleaned, a fluorescent sealant containing DAPI was added and the imaging was immediately observed under a laser confocal microscope.

### Western blotting assay

After the above operations were performed on PC12 cells, the supernatant was removed and RIPA lysis buffer was added to lysate the cells to obtain total cell protein. After detecting the protein concentration using the BCA protein assay kit, each group of proteins was diluted to the same concentration for use. The prepared protein was isolated using SDS-PAGE and then transferred to the activated polyvinylidene fluoride (PVDF) membrane. The PVDF membrane was incubated with 5% fat-free milk for 1h, and then incubated overnight with the corresponding primary antibody at 4 °C. After cleaning with TBST for 3 times, PVDF membrane was incubated with HPR-conjugated antibody (1:5,000) for 1 h. Finally, the membrane was imaged under enhanced chemiluminescence (ECL) system with Tublin as internal reference. Gray analysis of each blot was performed by the ImageJ software (version 1.51, National Institutes of Health, MD, United States).

### Determination of cell viability after the inhibition of signaling pathway

In this part, a chemical inhibitor LY294002 was used to inhibit the expression of PI3K/Akt signaling pathway to further clarify the role of the PI3K/Akt signaling pathway in the neuroprotection of AOF. In brief, the AOF group was incubated with 80 μg/ml AOF and 90 μM H_2_O_2_, the AOF + LY294002 group was pretreated with 20 μM LY294002 for 1 h and then incubated with 80 μg/ml AOF and 90 μM H_2_O_2_, while the LY294002 group was treated with only LY294002 and H_2_O_2_.

### Statistical analysis

Data are presented as mean ± standard deviations (SD). Statistical comparisons except the seizure rate were made by Student’s t-test or one-way analysis of variance (ANOVA) using GraphPad Prism five software (GraphPad Software Inc., La Jolla, CA). *p* < 0.05 was considered as the significant level.

## Results

### Compound identification of AOF and active component screening

**FIGURE 1:**
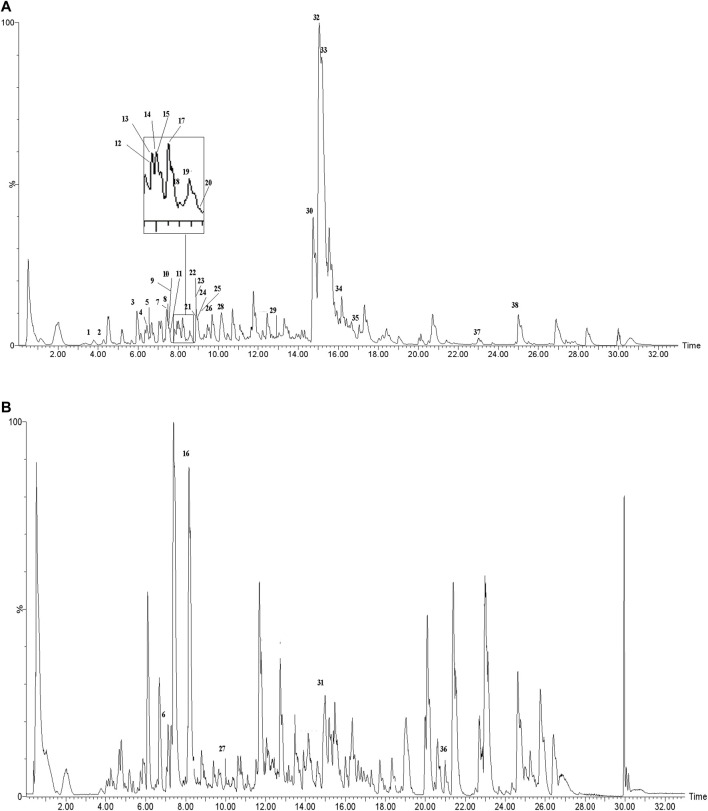
Total ion chromatogram monitored in positive **(A)** and negative **(B)** ion modes for AOF.

The actual ingredients of AOF were extracted by LC–MS and the results of the ion flow diagram are shown in Figure 1, while the actual chemical composition is shown in Table 1. As shown in Figure 1; Table 1, a total of 38 substances were obtained.

**TABLE 1:** Identified constituents of *Alpiniae oxyphyllae* Fructus by LC-MS.

No	t_R_/min	Compound	Formula	Structure
1	4.181	Oxyphyllenone A	C_12_H_18_O_3_	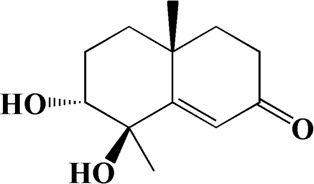
2	4.233	Oxyphyllenone B	C_12_H_18_O_3_	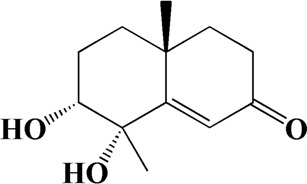
3	5.949	Oxyphyllenodiol A	C_14_H_22_O_3_	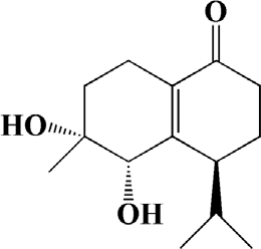
4	6.460	(11S)-Nootkatone-11,12-diol	C_15_H_24_O_3_	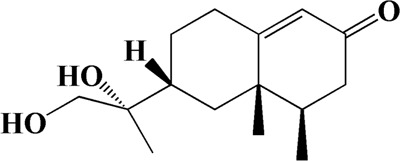
5	6.542	Oxyphyllanene B	C_12_H_14_O_2_	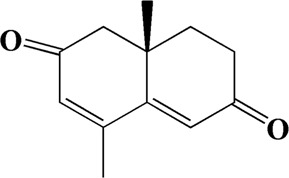
6	7.024	Protocatechuic acid	C_7_H_6_O_4_	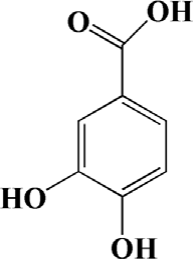
7	7.430	Oxyphyllone D	C_14_H_18_O_2_	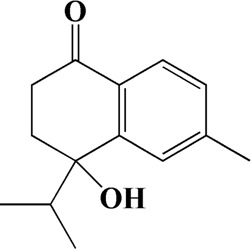
8	7.498	Oxyphyllone E	C_14_H_20_O_3_	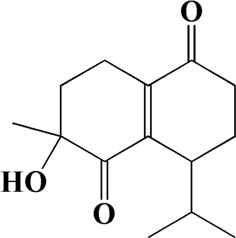
9	7.504	Cymol	C_10_H_14_	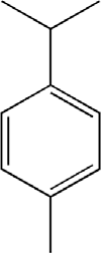
10	7.543	Teuhetenone A	C_12_H_18_O_2_	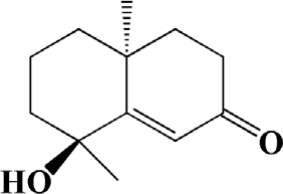
11	7.606	Oxyphyllenodiol B	C_14_H_22_O_3_	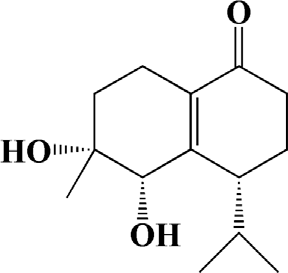
12	7.962	Oplopanone	C_15_H_26_O_2_	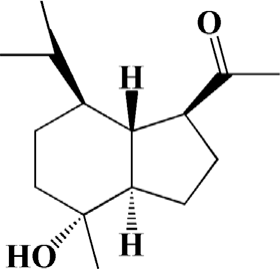
13	7.973	Oxyphyllanene A	C_12_H_16_O_2_	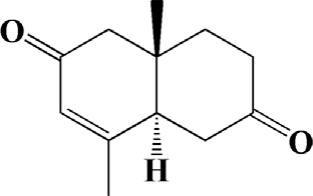
14	8.043	7-Epi-teucrenone	C_15_H_22_O_2_	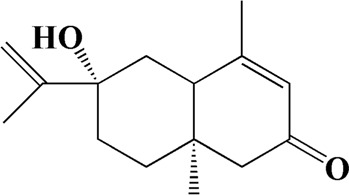
15	8.075	Teuhetenone B	C_12_H_18_O_2_	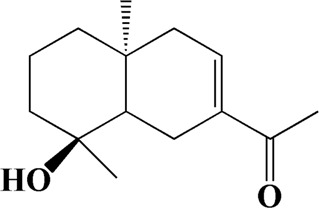
16	8.111	Beta-asarone	C_12_H_16_O_3_	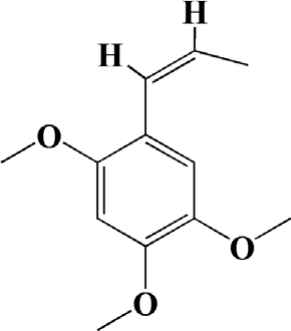
17	8.253	(5R,7S,10S)-5-hydroxy-13-noreudesma-4,6-dien-3,11-dione	C_14_H_20_O_3_	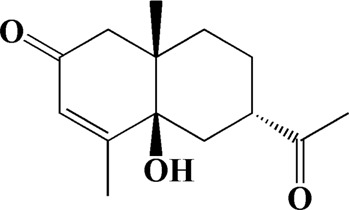
18	8.358	Oxyphyllone G	C_14_H_18_O_2_	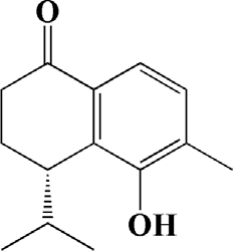
19	8.649	Diketone	C_14_H_20_O_2_	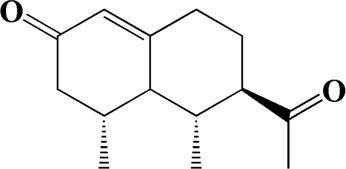
20	8.795	11-Hydroxy-valenc-1 (10)-en-2-one	C_15_H_24_O_2_	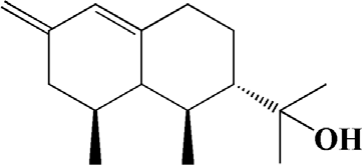
21	8.890	Oxyphyllol A	C_15_H_24_O	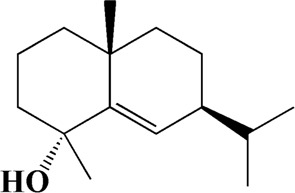
22	8.905	Nootkatol	C_15_H_24_O	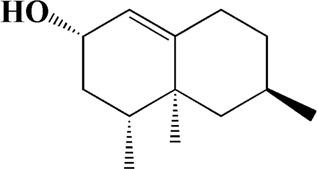
23	8.942	(4aS,7S)-7-hydroxy-1,4a-dimethyl-7-(prop-1-en-2-yl)-4,4a,5,6,7,8-hexahydronaphthalen2(3H)-one	C_15_H_22_O_2_	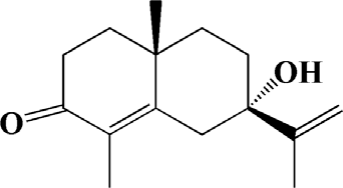
24	9.128	Eremophila-1(10),11(12)-dien-2,9-dione	C_15_H_20_O_2_	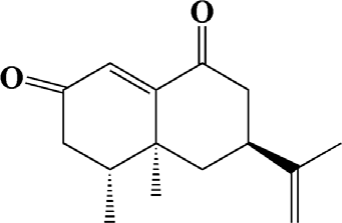
25	9.149	(E)-labda-12,14-dien-15 (16)-olide-17-oic acid	C_20_H_28_O_4_	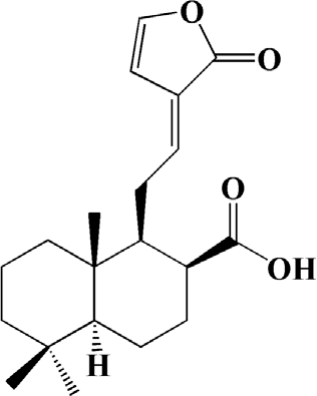
26	9.723	Dehydronootkatone	C_15_H_20_O	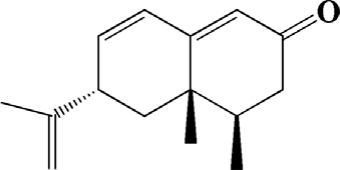
27	9.901	Rhamnocitrin	C_16_H_12_O_6_	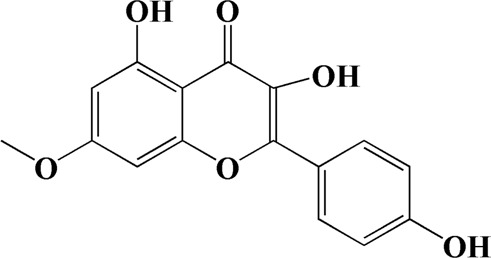
28	10.087	Oxyphyllenone H	C_14_H_22_O_2_	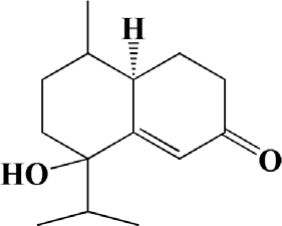
29	13.963	Gamma-Pinene	C_10_H_16_	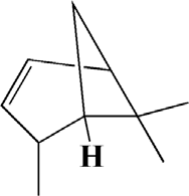
30	14.791	Tectochrysin	C_16_H_12_O_4_	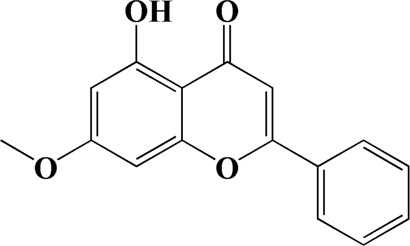
31	14.968	Yakuchinone b	C_20_H_22_O_3_	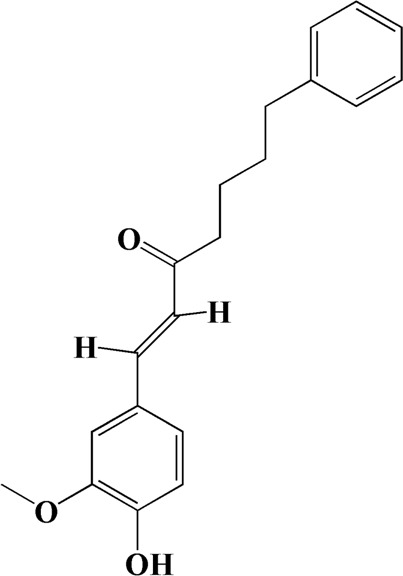
32	15.081	4-Isopropyl-6-methyltetralone	C_14_H_18_O	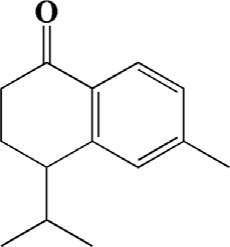
33	15.113	Nootkatone	C_15_H_22_O	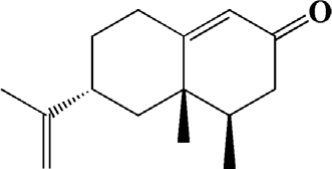
34	16.334	Yakuchinone-A	C_20_H_24_O_3_	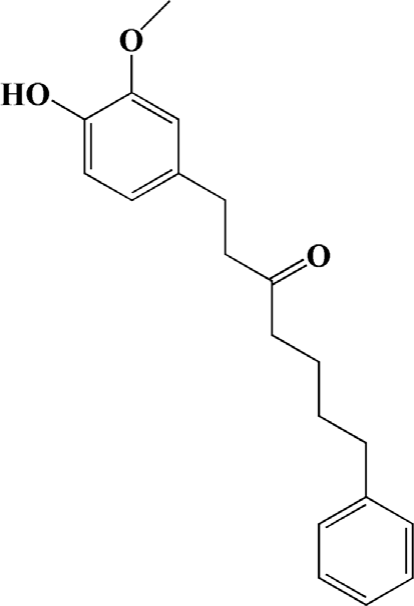
35	17.162	Alpha-Pinene	C_10_H_16_	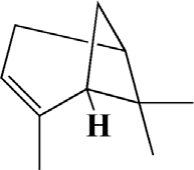
36	21.235	Linoleic acid	C_18_H_32_O_2_	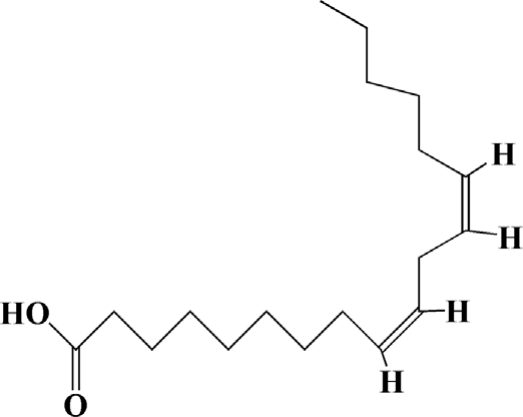
37	22.761	Valencene	C_15_H_24_	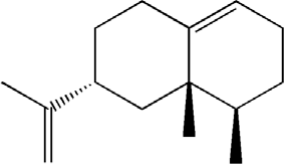
38	25.243	Stigmasterol	C_29_H_48_O	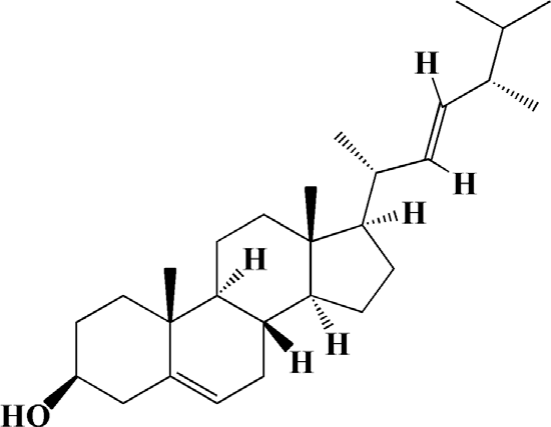

### Target prediction and network analysis of AOF against AD

**FIGURE 2:**
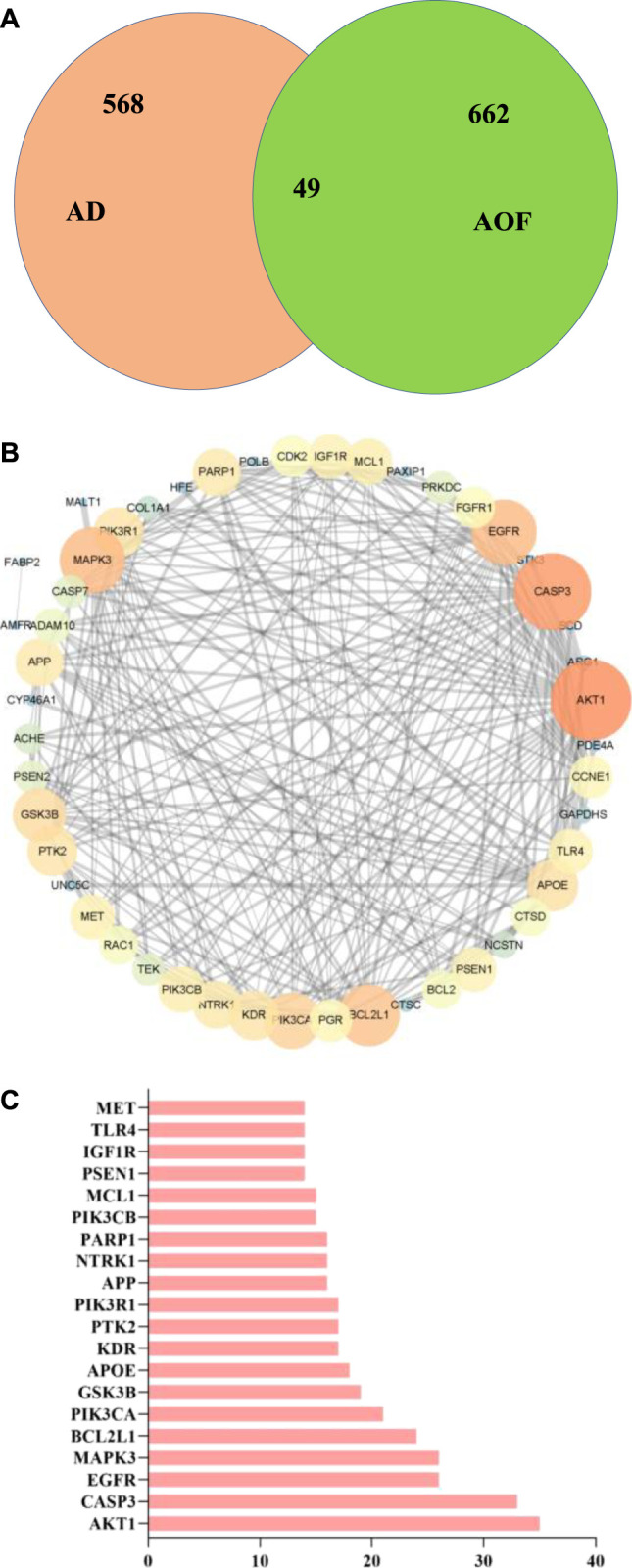
Overlapped targets screen **(A)** Venn diagram describing target distribution of AOF and AD **(B)** The protein-protein interaction (PPI) network of AOF target for the treatment of AD. The colors of the nodes are illustrated from orange to blue in descending order of degree values **(C)** The top 20 genes in the PPI network were selected based on the degree.

From online databases and previous reports, 662 targets of AOF were collected after deleting duplicated targets. On the other hand, we used OMIM and gene card database to screen AD genes and obtained 568 related target genes. Subsequently, the potential target genes of AOF and AD were intersected and obtained 49 overlapping genes, which were shown in the Venn diagram (Figure 2A). In others words, these 49 genes were considered the potential targets of AOF against AD.

Subsequently, we constructed PPI network through STRING database, and imported 49 overlapping genes into Cytoscape (ver.3.7.1) for visualization. The results are shown in Figure 2B, where each node represents a target protein and the edge represents the interaction between proteins. The most important 20 targets were selected according to node degree, as shown in Figure 2C, including AKT1, CASP3, EGFR, MAPK3, BCL2L1, PIK3CA, GSK3B, APOE, KDR, PTK2, PIK3R1, APP and so on, which means the main potential targets of AOF.

### Go and KEGG enrichment analyses

**FIGURE 3:**
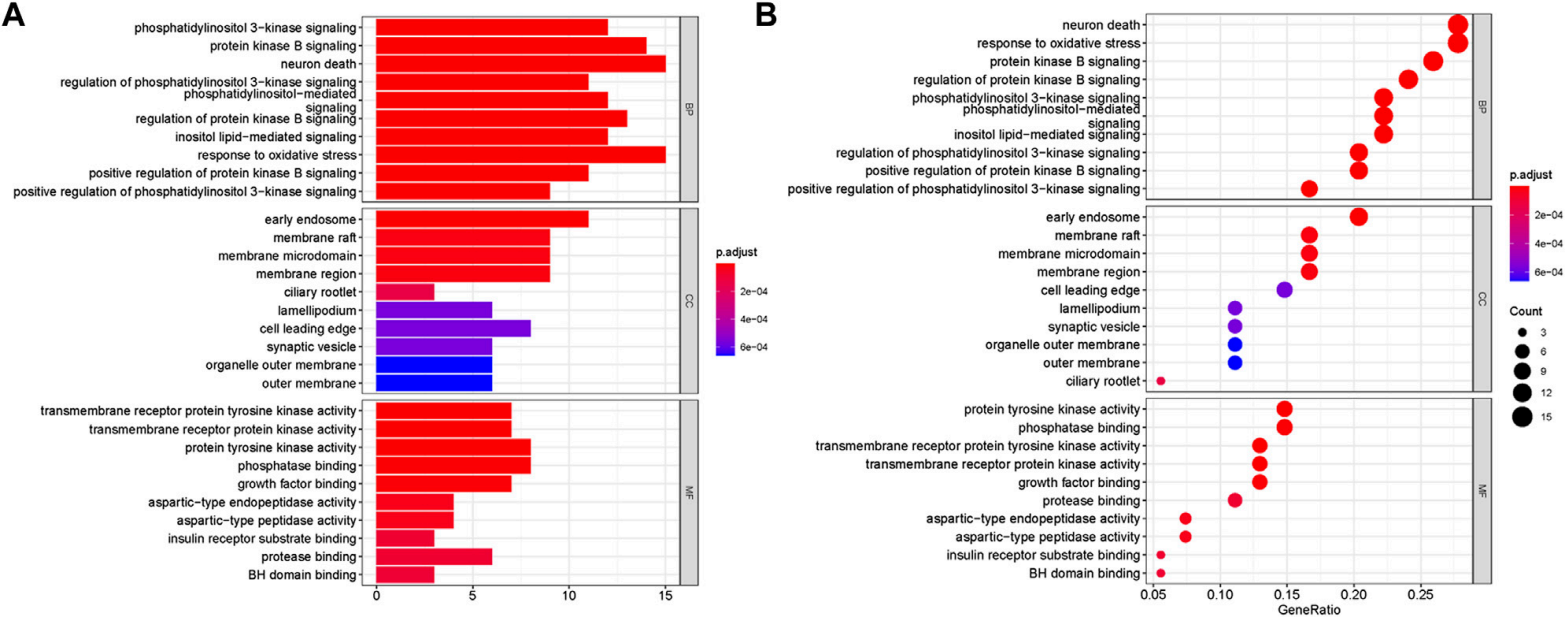
Go enrichment analyses of targets represented in bar chart **(A)** and bubble diagram **(B)**. The bubble size represents the number of genes enriched passage; the larger the bubble, the more enriched the genes. Bubble colors represent significant enrichment; the redder the color, the higher the degree of enrichment.

**FIGURE 4:**
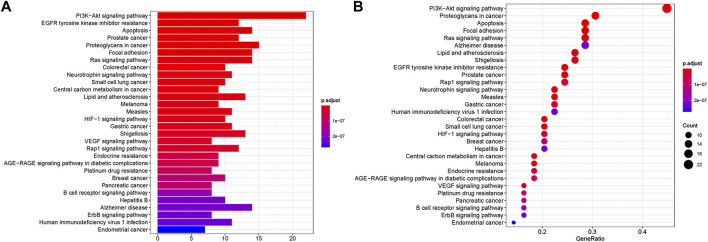
KEGG enrichment analyses of targets represented in bar chart **(A)** and bubble diagram **(B)**. The bubble size represents the number of genes enriched passage; the larger the bubble, the more enriched the genes. Bubble colors represent significant enrichment; the redder the color, the higher the degree of enrichment.

GO and KEGG analyses were performed on the above targets, and the results were shown in Figures 3, 4, where the *X*-axis indicated the degree of enrichment, and the color changes from blue to red, representing increased reliability. In GO analysis, biological processes (BPs), cellular components (CCs) and molecular functions (MFs) of the first ten items were ranked according to the *p*-values. The results were shown in Figure 3. The BPs of these potential targets were mainly response to oxidative stress, neuron death, protein kinase B signaling and phosphatidylinositol 3-kinase signaling. The CCs were related to early endosome, membrane raft and membrane microdomain. The main MFs are the protein tyrosine kinase activity, phosphatase binding, transmembrane receptor protein tyrosine kinase activity, transmembrane receptor protein kinase activity and growth factor binding.

Furthermore, KEGG analysis showed that 56 overlapping genes were enriched in multiple signaling pathways (Figure 4). In the bubble chart, we selected the top 30 enrichment pathways, and also expressed the enrichment degree of the gene by the *X*-axis, the enrichment amount of the gene by the bubble size, and the *p* value by the color depth. The main pathways of enrichment included PI3K-Akt signaling pathway, EGFR tyrosine kinase inhibitor resistance, apoptosis, prostate cancer, proteoglycans in cancer as well as Alzheimer disease. Among them, PI3K-Akt signaling pathway and apoptosis have been widely confirmed to be involved in oxidative stress-induced apoptosis in the development of AD, and this result was consistent with the key genes AKT1 and CASP3 related to apoptosis in PPI analysis. Therefore, these two results suggest that the anti-AD effect of AOF may be related to the inhibition of apoptosis induced by oxidative stress.

The Main Potential Active Component Against AD in AOF.

**FIGURE 5:**
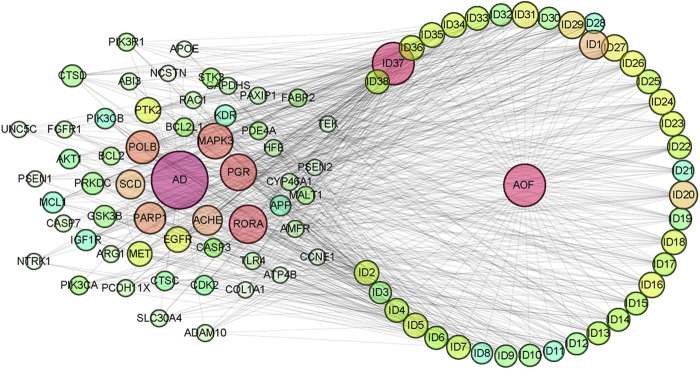
Network analysis of potential active compounds in AOF and AD targets. The drug, active compounds, disease and targets were input into Cytoscape to construct the DMTD diagram, and the colors of the nodes are illustrated from red to green in descending order of degree values (ID1: Protocatechuic acid; ID2: Oxyphyllanene A; ID3: Oxyphyllenodiol A; ID4: Oxyphyllenone A; ID5: Oxyphyllenone B; ID6: (11S)-Nootkatone-11,12-diol; ID7: Oxyphyllanene B; ID8: Oxyphyllone E; ID9: Oxyphyllone D; ID10: Teuhetenone A; ID11: Cymol; ID12: Oxyphyllenodiol B; ID13: Oplopanone; ID14: 7-Epi-teucrenone; ID15 (5R,7S, 10S)-5-hydroxy-13-noreudesma-4,6-dien-3,11-dione; ID16: Oxyphyllone G; ID17: Teuhetenone B; ID18: Diketone; ID19: 11-Hydroxy-valenc-1 (10)-en-2-one; ID20: Rhamnocitrin; ID21: Dehydronootkatone; ID22: Beta-asarone; ID23: Nootkatol; ID24: Oxyphyllol A; ID25: Oxyphyllenone H; ID26 (4aS, 7S)-7-hydroxy-1,4a-dimethyl-7-(prop-1-en-2-yl)-4,4a, 5,6,7,8-hexahydronaphthalen2(3H)-one; ID27: ID27: Eremophila-1(10),11(12)-dien-2,9-dione; ID28 (E)-labda-12,14-dien-15 (16)-olide-17-oic acid; ID29: Tectochrysin; ID30: Yakuchinone b; ID31: Yakuchinone-A; ID32: 4-Isopropyl-6-methyltetralone; ID33: Nootkatone; ID34: Gamma-Pinene; ID35: Alpha-Pinene; ID36: Valencene; ID37: Linoleic acid; ID38: Stigmasterol).

After importing 38 compounds in AOF and 56 targets intersecting with AD into Cytoscape, Figure 5 is obtained, which contains 94 nodes and 687 edges. In Figure 5, the color changes from blue to red as the number of connections increases. It can be clearly seen from Figure 5 that 38 components acted on a variety of targets, indicating that AOF has a multi-component and multi-target effect on AD. According to DMTD and previous studies, we selected protocatechuic acid and nootkatone, the key active ingredients of AOF, as the potential active ingredients of AOF against AD, and used them in subsequent experimental studies (He et al., 2018; Choi et al., 2020).

### Experimental validation *in vitro*

#### Protective effect of AOF on cell viability

**FIGURE 6:**
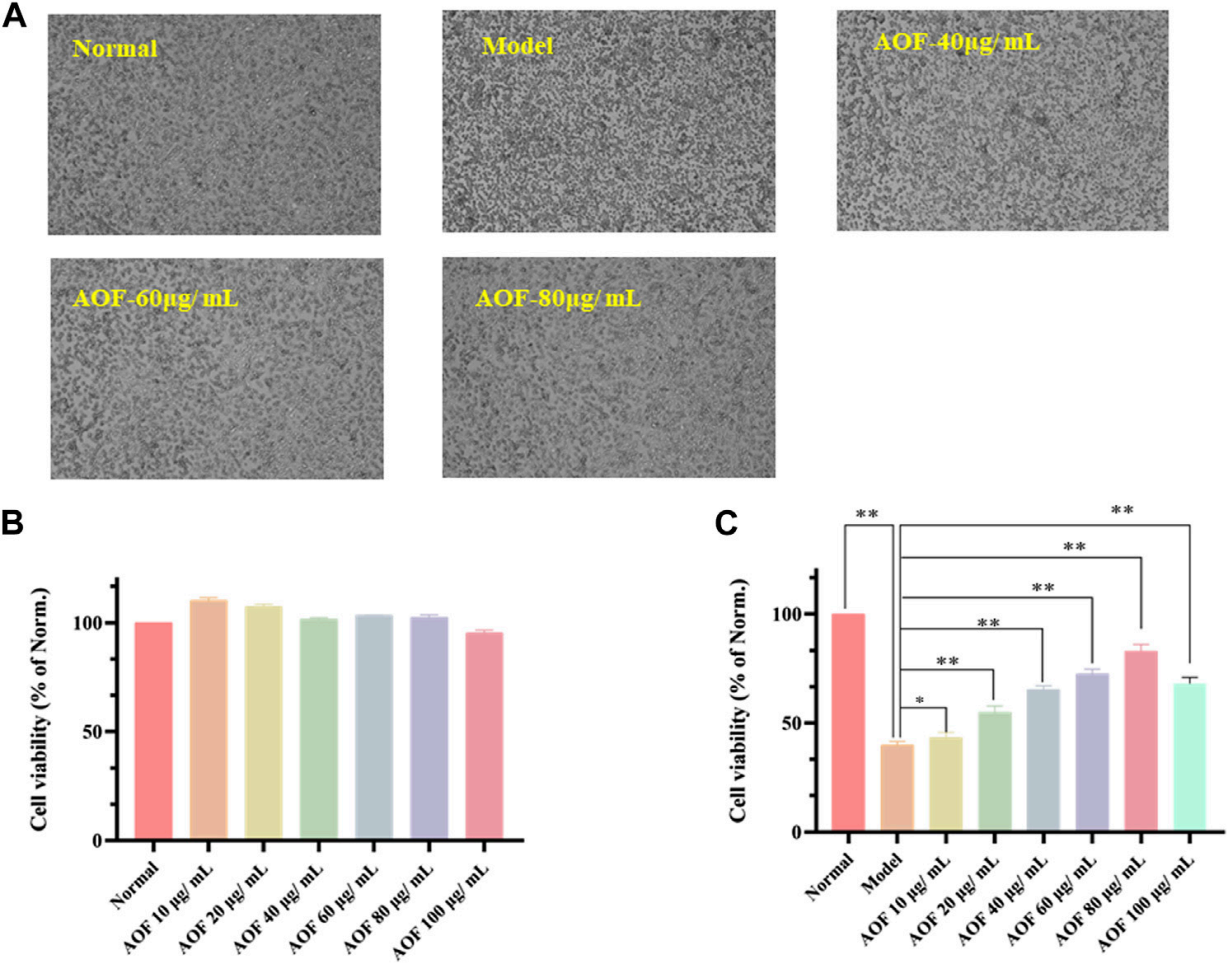
Protective effects of AOF on the cell viability of H_2_O_2_-stimulated PC12 cells **(A)** The represented cell morphology of PC12 cells with different treatment **(B)** Effects of AOF on cell viability of normal PC12 cell **(C)** Effects of AOF on cell viability of H_2_O_2_-stimulated PC12 cells under different concentration. The values were represented as the mean ± SD (*n* = 3). *p < 0.05, **p < 0.01 vs the model group.

As shown in Figure 6B, 10, 20, 40, 60 and 80 μg/ml AOF showed no significant toxicity to cells when incubated with PC12 cells (*p* < 0.05), while 100 μg/ml AOF had a little toxicity. In addition, the cell viability decreased to about 39.85% of the normal group when PC12 cells were selected to incubated with 90 μM H_2_O_2_ for 4 h (*p* < 0.01). At the same time, the number of adherent cells decreased significantly, while the cell morphology was smaller than that of normal cells (Figures 6A,C). In subsequent experiments, PC12 cells were pretreated with different concentrations of AOF (10, 20, 40, 60 80 and 100μg/ml) for 2 h and then stimulated with 90 μM H_2_O_2_ for 4 h. According to the experimental results, 10–100ug/mL AOF could increase cell viability to 43.38, 54.93, 65.44, 72.65, 83.08 and 68.17%. These results suggested that the protective effect of 10 ug/mL AOF on cell viability was not significantly different from that of model group, while the protective effect of 20-80ug/mL AOF increased in a dose-dependent manner. In addition, the protective effect of 100ug/mL AOF was slightly decreased. Combined with the results in Figure 6B, it was speculated that 100ug/mL AOF might be slightly toxic to PC12 cells. Considering the above results, 40, 60 and 80ug/mL AOF were selected as experimental concentrations in subsequent experiments.

### AOF suppressed apoptosis in H_2_O_2_-stimulated PC12 cells

**FIGURE 7:**
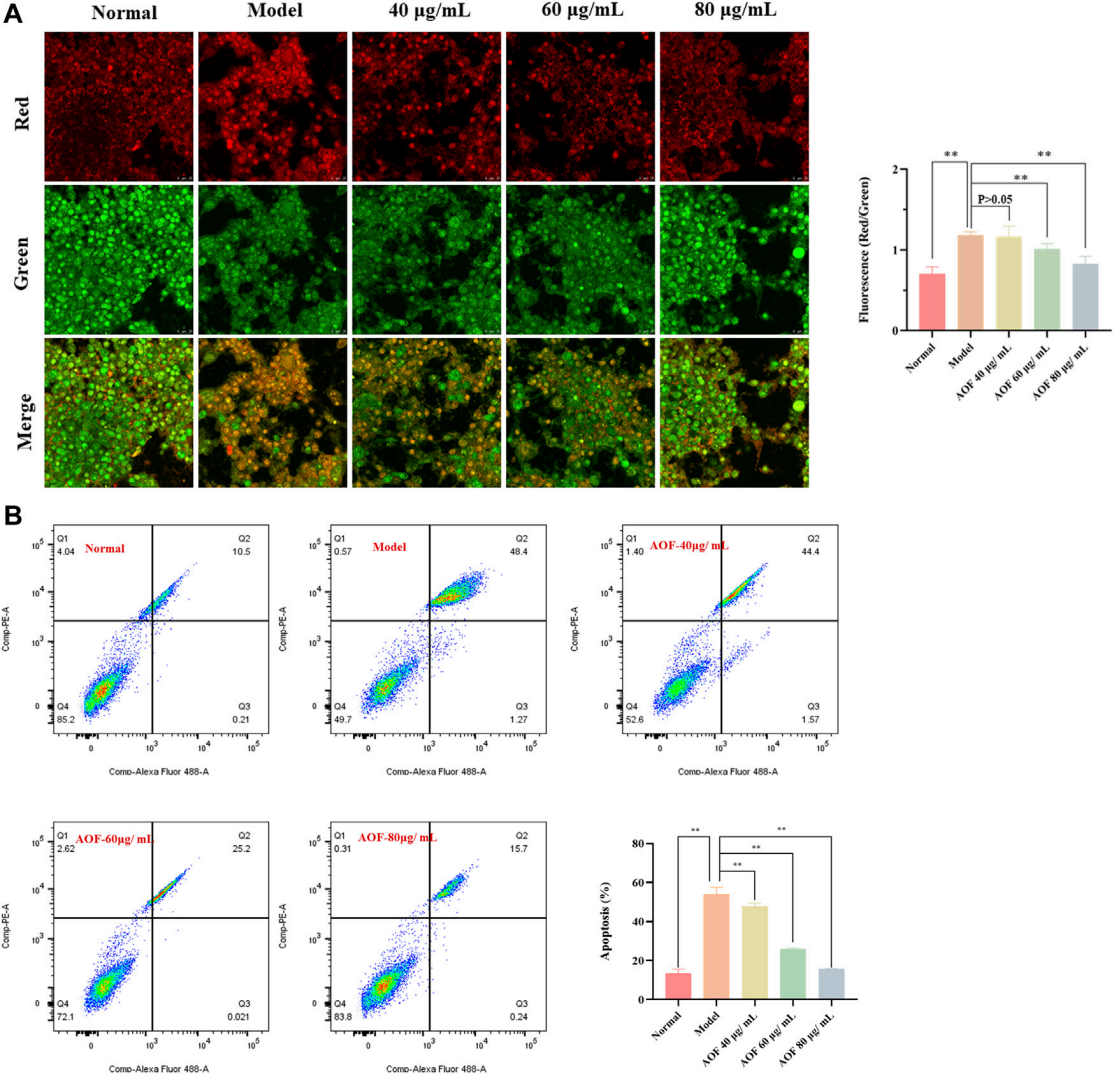
Effects of AOF on apoptosis in H_2_O_2_-stimulated PC12 cells **(A)** Apoptotic assay by AO/EB staining **(B)** Apoptotic assay by flow cytometry. The values were represented as the mean ± SD (*n* = 3). *p < 0.05, **p < 0.01 vs the model group.

Firstly, AO/EB staining was used to observe the permeability of plasma membrane of PC12 cells. When the cell is normally active, its membrane is intact. In other words, AO can intercalate into nuclear DNA through the living cell membrane and excite bright green fluorescence. Therefore, AO/EB staining is often used to distinguish apoptotic cells from normal cells. As shown in Figure 7A, when cells were stimulated with 90 μM H_2_O_2_, the intensity of red fluorescence in the model group increased sharply and the intensity of green fluorescence decreased as compared with the normal group. At the same time, after pretreatment with different concentrations of AOF (40, 60 and 80 μg/ml), the above situation was reversed to varying degrees, that is, compared with the model group, the red fluorescence intensity of the drug treatment group decreased, while the green fluorescence intensity increased. This result preliminarily suggested that AOF might inhibit H_2_O_2_-induced apoptosis of PC12 cells.

To further confirm the inhibitory effect of AOF on apoptosis, we then used Annexin V/PI staining kit to quantitatively analyze the apoptosis of PC12 cells. The experimental results are shown in Figure 7B. After stimulation with 90 μM H_2_O_2_, the apoptosis rate of PC12 cells increased to about 53.53%, which was significantly higher than that in the normal group (*p* < 0.01). At the same time, compared with the model group, pretreatment with different concentrations of AOF (40, 60 and 80 μg/ml) significantly reduced apoptosis (*p* < 0.01), and the apoptosis rate of 80 μg/ml AOF pretreatment was close to normal group.

**FIGURE 8:**
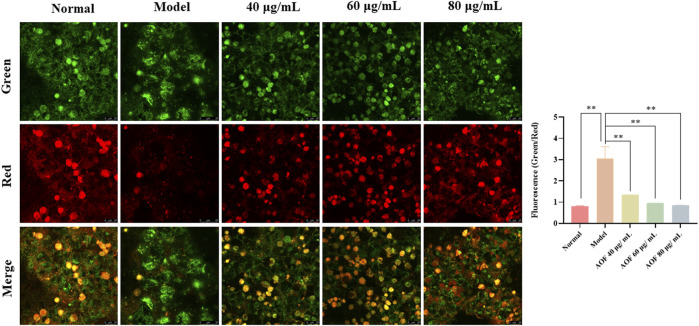
Effects of AOF on the ΔΨm in H_2_O_2_-stimulated PC12 cells (×40). ΔΨm was measured using a JC-1 assay kit and observed using a laser confocal microscopy. When mitochondrial function is normal, the MMP is at a higher potential, and JC-1 can form a polymer that aggregate in the mitochondrial matrix and emit red fluorescence. However, when it is at the depolarized membrane potential, JC-1 could only exist in the cytoplasm as a monomer and emit green fluorescence. Data were expressed as the mean ± SD (*n* = 3). **p < 0.01 vs model group.

Subsequently, we detected MMPs using the JC-1 fluorescent probe. When mitochondrial function is normal, the MMP is at a higher potential, and JC-1 can form a polymer that aggregate in the mitochondrial matrix and emit red fluorescence. However, when it is at the depolarized membrane potential, JC-1 could only exist in the cytoplasm as a monomer and emit green fluorescence **(** Li et al., 2020 **)**. Therefore, the ratio of red and green fluorescence emitted by JC-1 after incubation with cells can reflect the level of MMP, indicating whether the mitochondrial physiological function is normal. In addition, the decline of MMP is also widely recognized as an early event in the occurrence of apoptosis. The detection results are shown in Figure 8. After H_2_O_2_ stimulation, the green fluorescence intensity in the PC12 cells increased and the red fluorescence intensity decreased sharply. The green/red fluorescence ratio increased significantly (*p* < 0.01). However, after pretreatment with different concentrations of AOF, the above situation was improved, indicating that AOF has a protective effect on MMPs stimulated by H_2_O_2_.

### Effects of main active components on cell viability and apoptosis

**FIGURE 9:**
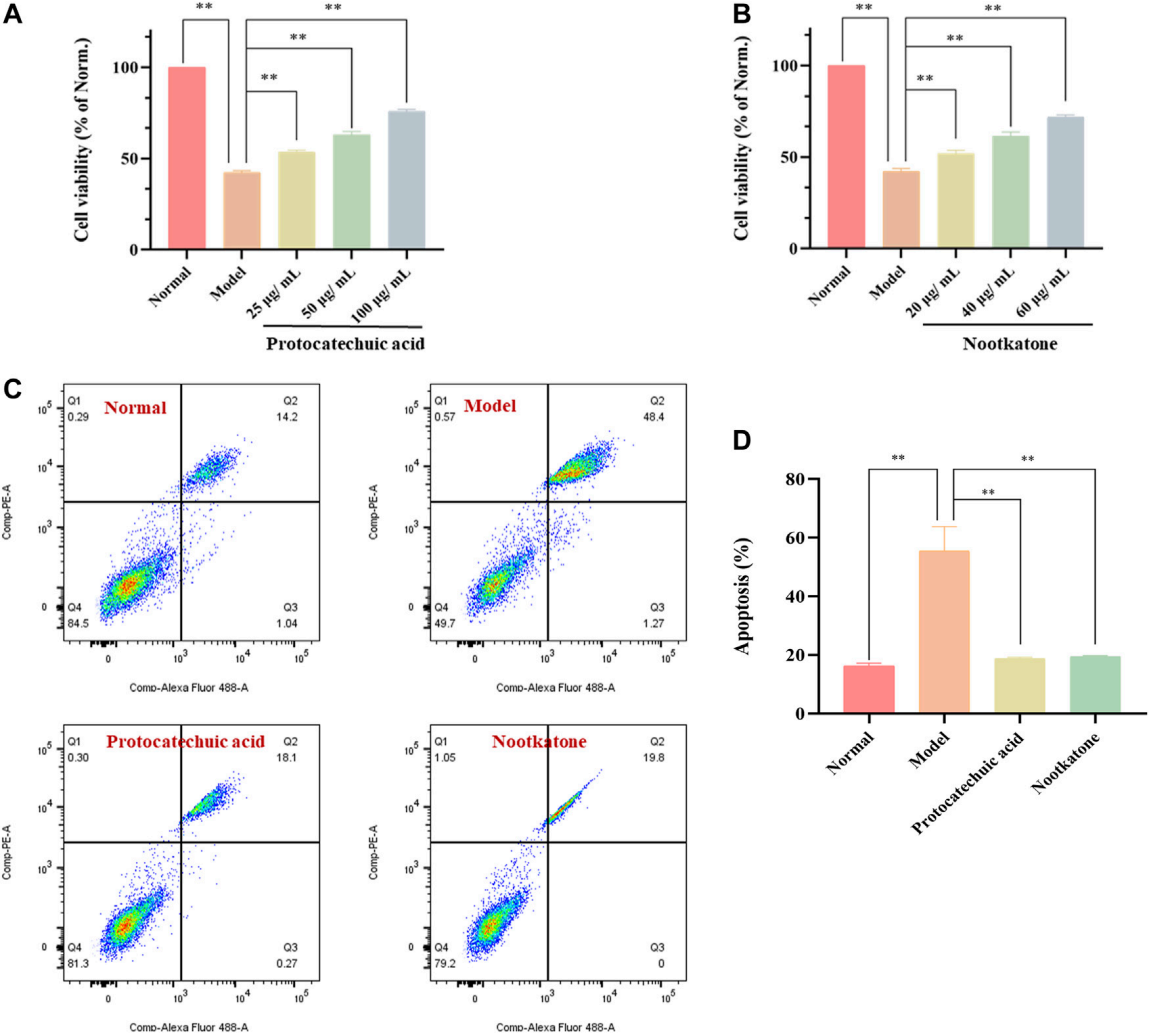
Effects of the main active components (protocatechuic acid and nootkatone) on cell viability and apoptosis in H_2_O_2_-stimulated PC12 cells **(A)** Effects of protocatechuic acid on cell viability of H_2_O_2_-stimulated PC12 cells under different concentration (25, 50 and 100 μg/ml) **(B)** Effects of nootkatone on cell viability of H_2_O_2_-stimulated PC12 cells under different concentration (20, 40 and 60μg/ml) **(C)** Apoptotic assay by flow cytometry **(D)** Apoptosis ratio statistics. The values were represented as the mean ± SD (*n* = 3). **p < 0.01 vs model group.

The effects of the two components (protocatechuic acid and nootkatone) on cell viability and apoptosis were shown in Figure 9. The results showed that compared with the model group, the two active ingredients increased the activity of PC12 cells stimulated by H_2_O_2_ to different degrees. Among them, 25, 50 and 100μg/ml protocatechuic acid could increase the cell viability to 53.5, 62.9 and 75.8% of the normal group, respectively, while 20, 40 and 60μg/ml nootkatone could increase the cell viability to 52.0, 61.4 and 71.8% of the normal group (*p* < 0.01) (Figures 9A,B). Subsequently, apoptosis was analyzed using Annexin V/PI staining kit. The results showed that both active ingredients could inhibit the apoptosis induced by H_2_O_2_ stimulation to varying degrees. Among them, 100μg/ml protocatechuic acid could inhibit the cell apoptosis at 18.74%, while 60μg/ml nootkatone could control the cell apoptosis at 19.40% (*p* < 0.01) (Figures 9C,D).

### AOF decreases ROS generation in H_2_O_2_-stimulated PC12 cells

**FIGURE 10:**
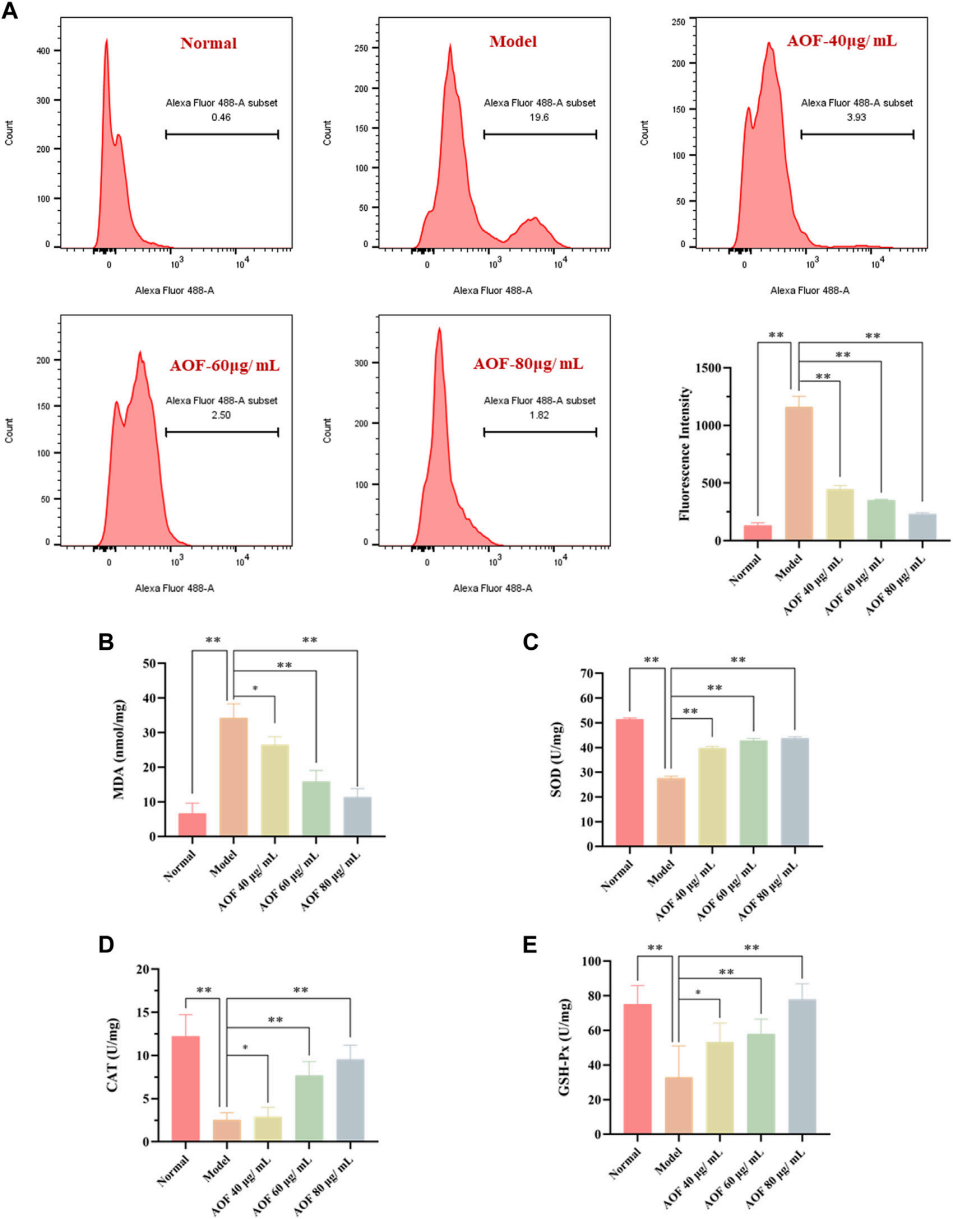
Effects of AOF on ROS levels **(A)** and antioxidant enzyme activities **(B–E)** in H_2_O_2_-stimulated PC12 cells **(A)** The intracellular ROS levels were measured using a DCFH-DA assay kit and flow cytometry **(B–E)** The levels of MDA and activities of SOD, CAT and GSH-Px were determined by commercial assay kits. The values were represented as the mean ± SD (*n* = 3). *p < 0.05, **p < 0.01 vs the model group.

Previously, it has been confirmed that AOF could inhibit the apoptosis of PC12 cells induced by H_2_O_2_ and also protect MMP. As is known to all, cells stimulated by H_2_O_2_ will produce a large number of ROS, and the excess of ROS is also an important inducement of apoptosis. Therefore, in order to further explore the mechanism behind the protective effect of AOF, we further used DCFH-DA to detect intracellular ROS. As shown in Figure 10A, when PC12 cells were exposed to H_2_O_2_, intracellular ROS content increased sharply. At the same time, compared with the model group, the content of ROS decreased to different degrees after pretreatment with different concentrations of AOF (40, 60 and 80μg/ml), in a dose-dependent manner (*p* < 0.01).

When oxidative stress occurs, cells will produce excessive MDA. At the same time, ROS scavenging enzymes such as SOD, CAT and GSH-Px, which are important regulators of redox balance, may also decrease. To further investigate whether AOF plays an anti-apoptotic role by protecting cells from oxidative stress injury, we further examined these intracellular biomarkers that can be used to assess cellular oxidative stress. The results are shown in Figures 10B–E. When stimulated by H_2_O_2_, MDA in PC12 cells increased to 34.19 nmol/mg, while SOD, CAT and GSH-Px contents decreased to 27.58 U/mg, 2.54 U/mg and 32.77 U/mg, respectively (*p* < 0.01). Surprisingly, these conditions were improved after pretreatment with different concentrations of AOF. Among them, 60 and 80 μg/ml AOF can reduce MDA to 15.92 U/mg and 11.38 U/mg (*p* < 0.01), while 40 μg/ml AOF has no obvious effect on MDA (*p* < 0.05). At the same time, 60 and 80 μg/ml AOF could increase CAT and GSH-Px contents (*p* < 0.01), while 40 μg/ml AOF had no significant difference in CAT and GSH-Px compared with model group (*p* < 0.05). In addition, different concentrations of AOF (40, 60 and 80 μg/ml) could significantly increase the content of SOD, respectively (*p* < 0.01). These results suggest that AOF can protect cells from oxidative stress damage.

### Molecular mechanism of the protective effects of AOF in H_2_O_2_-stimulated PC12 cells

**FIGURE 11:**
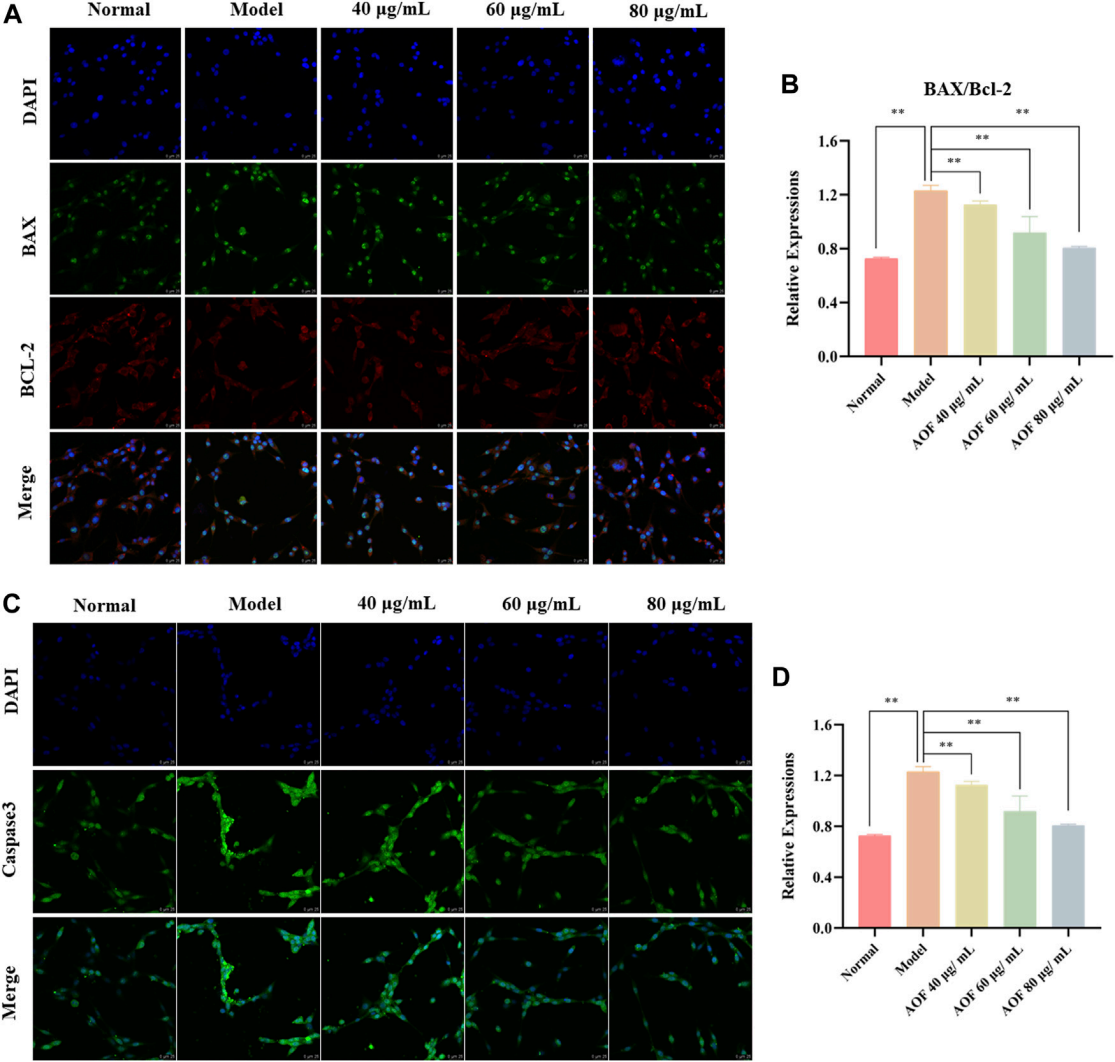
Effects of AOF on apoptosis proteins in H_2_O_2_-stimulated PC12 cells **(A)** IF images of Bcl-2 and Bax **(B)** Analysis of the Bcl-2/Bax ratio **(C)** IF images of Caspase-3 **(D)** Analysis of the Caspase-3. Data were expressed as mean ± SD (*n* = 3), **p < 0.01 vs model.

According to the GO and KEGG analyses, the protective effect of AOF may be related to apoptosis induced by oxidative stress, and may also be related to PI3K/Akt signaling pathway. Therefore, in this part of the experiment, immunofluorescence was used to explore the molecular mechanism of the anti-apoptotic effect of AOF on H_2_O_2_-stimulated PC12 cells. Bcl-2 and BAX, as anti-apoptotic protein and pro-apoptotic protein respectively, are key regulators of mitochondria mediated apoptosis, while caspase3 is an important executor of apoptosis. Therefore, we used immunofluorescence to observe the expression of these three proteins in cells, and found that the expression of BAX and caspase3 was significantly increased after H_2_O_2_ stimulation, while the expression of anti-apoptotic protein Bcl2 was inhibited (*p* < 0.01). At the same time, pretreatment with different concentrations of AOF could reverse this change (*p* < 0.01) (Figure 11).

**FIGURE 12:**
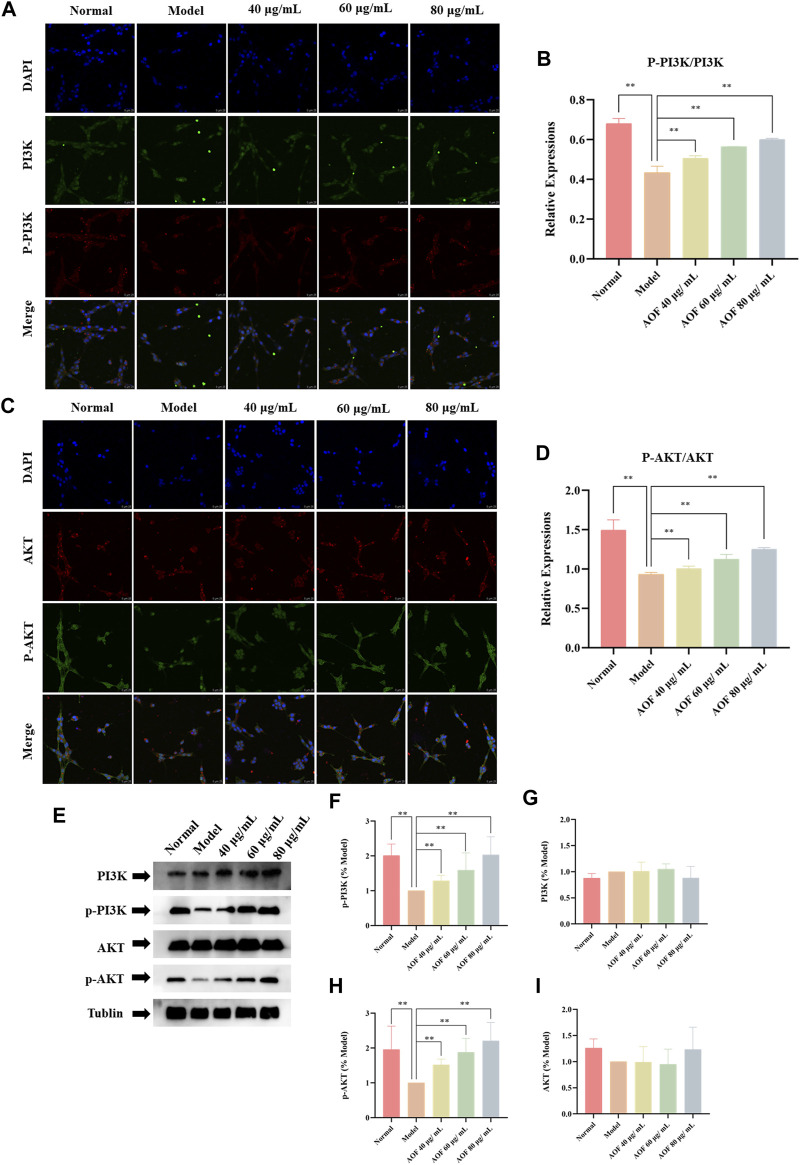
Effects of AOF on PI3K/Akt signaling pathway in H_2_O_2_-stimulated PC12 cells **(A)** IF images of p-PI3K and PI3K **(B)** Analysis of the p-PI3K/PI3K ratio **(C)** IF images of p-Akt and Akt **(D)** Analysis of the p-Akt/Akt ratio **(E)** WB images of p-Akt, p-PI3K, AKT and PI3K protein expression **(F)** Analysis of P-PI3K **(G)** Analysis of PI3K **(H)** Analysis of P-Akt **(I)** Analysis of Akt. Data were expressed as mean ± SD (*n* = 3), *p < 0.05, **p < 0.01 vs model.

Subsequently, the expression of PI3K, P-PI3K, Akt and P-Akt in cells was also detected by immunofluorescence. The results showed that there was no significant difference in the expression of PI3K and AKT in all groups. In the model group, the contents of p-PI3K and p-Akt were significantly decreased (*p* < 0.01), while the expressions of p-PI3K and p-Akt were increased to varying degrees in the AOF pretreatment group (*p* < 0.01), indicating that AOF can activate the PI3K/AKT signaling pathway (Figures 12A–D). The same results were found in WB, which were shown in Figures 12E,F. The WB results showed that there was no difference in the protein expression of PI3K and Akt in all groups. The protein expression of P-PI3K and P-Akt were decreased when cells were stimulated by H_2_O_2_, and AOF pretreatment could reverse these situations (*p* < 0.01).

**FIGURE 13:**
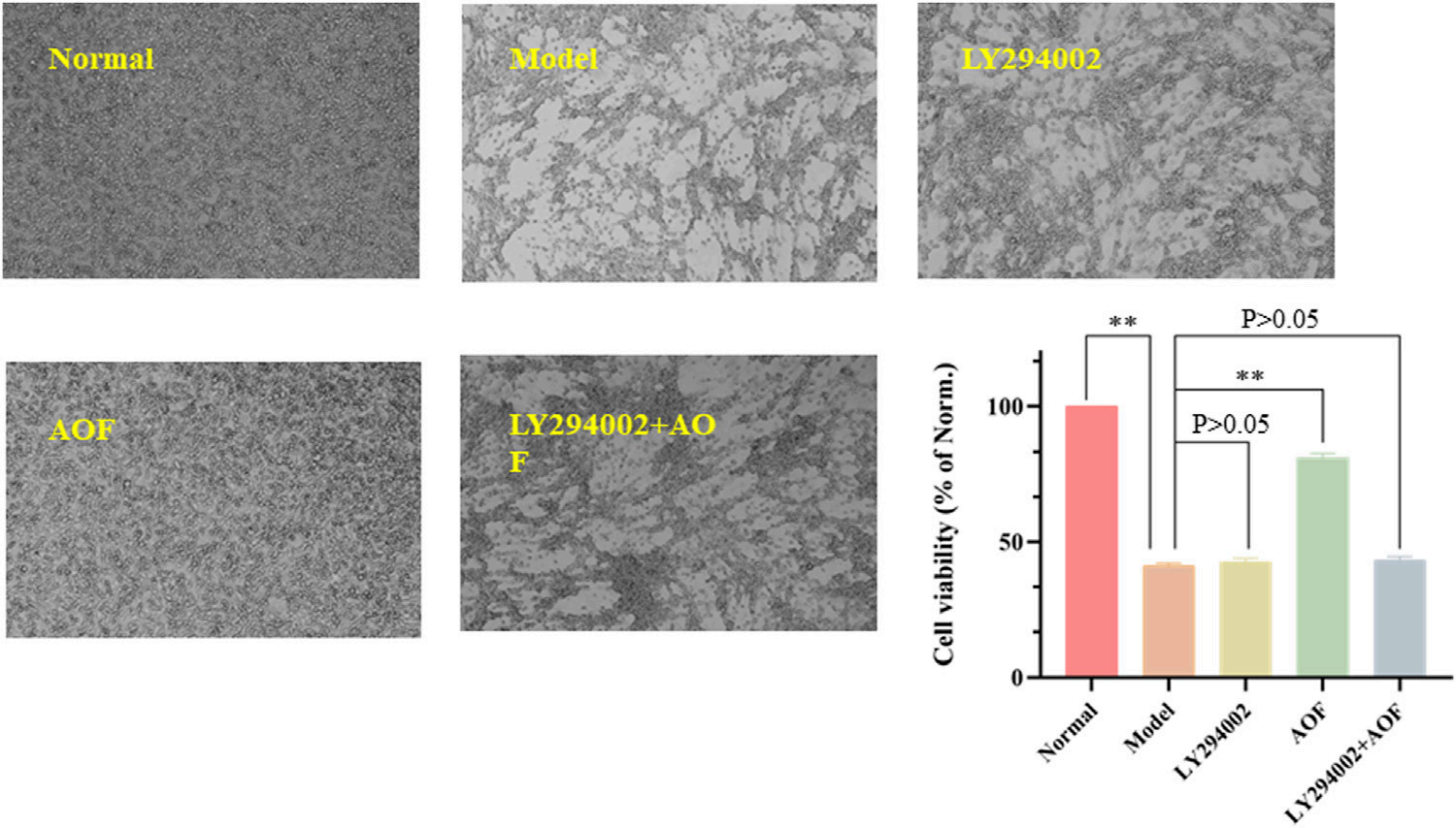
Effects of PI3K inhibitor LY294002 on the viability of PC12 cells. The PC12 cells were pretreated with LY294002 (20 μM) or not for 1 h; then cells were treated with AOF (80 μg/ml) for 2 h, subsequently subjected to H_2_O_2_ for 4 h. The values were represented as the mean ± SD (*n* = 3). ∗∗*p* < 0.01 vs. the model group.

In order to further investigate whether the PI3K/AKT signaling pathway is the key signaling pathway for AOF to protect PC12 cells from H_2_O_2_ stimulation, we subsequently used a chemical inhibitor LY294002, the first synthetic small molecule to inhibit PI3K, to decrease the expression of the PI3K/AKT signaling pathway. Results were shown in Figure 13, cell viability decreased to 40.5% after H_2_O_2_ stimulation. When treated with LY294002 alone, the viability of PC12 cells was not significantly different from that of the model group, which was about 42.7% (*p* > 0.05). In addition, cell viability was significantly improved when treated with AOF (*p* < 0.01). However, when treated with AOF and LY294002, cell viability was only slightly increased, and there was no significant difference compared with model group (*p* > 0.05). These results suggest that AOF protects PC12 cells from H_2_O_2_-induced apoptosis through PI3K/AKT pathway.

## Discussion

In the past decades, as a neurodegenerative disease, the incidence of AD has increased year by year, threatening more and more elderly population (Li et al., 2021; Yao et al., 2022). As a natural medicine, AOF contains a variety of components and has the advantage of multiple targets, which also brings great challenges to researchers (Zuo et al., 2021; Peng et al., 2022). In this study, we combined network pharmacology and related *in vivo* experimental to explore the protective effect of AOF on AD. First, 39 components contained in the AOF were identified by LC-MS and then imported into the TCMSP and SwissTarget online databases. As a result, 662 targets were predicted. Meanwhile, we also collected 568 AD-related targets from OMIM and Gene card databases. After taking the intersection of the above two targets and performing the corresponding GO and KEGG analysis, we could find that the anti-AD effect of AOF may be related to the inhibition of apoptosis caused by oxidative stress, while protocatechuic acid and nootkatone in AOF may be the main active ingredient for protection. In PPI network analysis, it was found that the targets of AOF including apoptosis proteins such as AKT1, caspase3 and Bcl-2, and their role in apoptosis cannot be ignored. In addition, in the currently studies, the academic community generally believes that excessive apoptosis of nerve cells is an important inducement for AD. From the analysis results of GO, we could know that the anti-AD effect of AOF may be related to oxidative stress. It is well known that the ability of cells to scavenge ROS has a certain limit. When the content of ROS in nerve cells exceeds this limit, the cells may undergo oxidative stress. Oxidative stress in turn not only exacerbates the excessive accumulation of ROS, but also promotes apoptosis. Finally, a conclusion was drawn in the KEGG analysis that AOF may play a protective role through the PI3K/AKT signaling pathway.

It has been confirmed in recent experiments that the PI3K/AKT signaling pathway is one of major signaling cascades in cell survival and development as well as in ROS-induced apoptosis. The activation of this pathway can also recruit PI3K to the plasma membrane, where PI3K interacts with Akt resulting in phospholipid modifications (Shaw and Cantley, 2006). A direct consequence of PI3K phosphorylation can lead to phosphorylation of AKT, while the full activation of Akt requires its phosphorylation at two distinct sites (Guertin et al., 2006). The principal role of Akt in anti-apoptotic signaling has been established by many researchers in different cell death paradigms. Mechanistically, BAX and Bcl2, as downstream proteins of AKT, can be regulated by AKT. When AKT is activated, BAX expression is decreased and Bcl2 expression is increased, thereby exerting an anti-apoptotic effect (Bender et al., 2011; Kapodistria et al., 2015). In addition, it has been confirmed in multiple studies that the PI3K/Akt signaling pathway plays a major role in redox homeostasis by inhibiting the production of ROS (Hu et al., 2018; Li et al., 2018). It is well-known that excessive ROS will lead to a decrease in MMP and an increase in mitochondrial membrane permeability, resulting in cytochrome C (Cyt-C) efflux. Subsequently, Cyt-C in the cytoplasm activates caspase-9, which in turn activates Caspase-3. As the ultimate executor of apoptosis, caspase-3 activation triggers the occurrence of apoptosis (Zhang et al., 2021).

**FIGURE 14:**
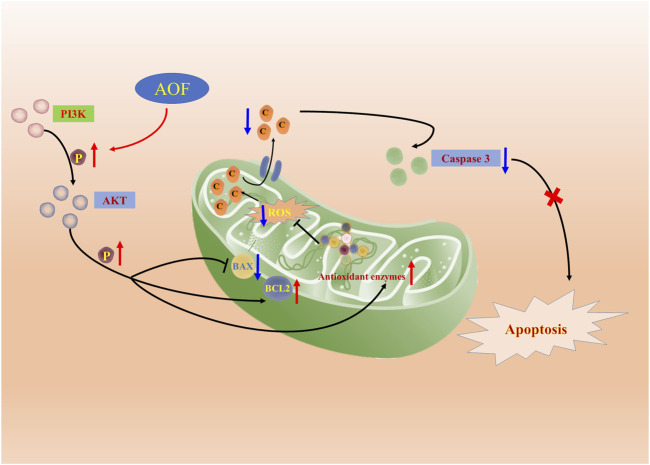
Molecular mechanism of the protective effect of AOF. AOF possesses protective potentials on H_2_O_2_-stimulated PC12 cells through suppression of oxidative stress-induced apoptosis via regulation of the PI3K/Akt signal pathway.

In order to verify whether the hypothesis proposed in the above network analysis is correct, we then conducted corresponding *in vitro* experiments to further verify. PC12 is a rat-derived cell line with similar characteristics to neurons, which can mimic the physiological and pathological processes of nerve cells (Lant et al., 2021; Chen et al., 2022). In addition, H_2_O_2_ is a small molecule compound with high cell membrane permeability. At the same time, as an important member of the ROS family, H_2_O_2_ can also generate hydroxyl radicals, which in turn cause oxidative stress and even cell death (Zhang et al., 2021). Therefore, PC12 cells stimulated by H_2_O_2_ are often used in drug screening models for various neurodegenerative diseases, and this model was also selected as a cell model to verify the anti-AD effect of AOF in this experiment (Bie et al., 2021; Vahdati et al., 2022). Experiments verified that the viability of PC12 cells decreased to 40% after incubation with 90 μM H_2_O_2_, while different concentrations of AOF, protocatechuic acid and nootkatone could restore cell viability to varying degrees. Next, we detected the apoptosis in various ways, and found that H_2_O_2_ would increase the apoptosis of PC12 cells and decrease the mitochondrial membrane potential, while AOF pretreatment effectively inhibited the apoptosis of PC12 cells and increased the mitochondrial membrane potential. More importantly, in subsequent experiments, we also confirmed that AOF could reduce the content of ROS and MDA in PC12 cells stimulated by H_2_O_2_, and increase the content of three oxidoreductases, SOD, GSH-Px and CAT in the cells. In this way, it was verified that AOF could reduce apoptosis by inhibiting cellular oxidative stress. Finally, in cell immunofluorescence, we also found that AOF increased the expression of anti-apoptotic protein Bcl2, while the expression of pro-apoptotic proteins BAX and caspase3 decreased. On the one hand, after detecting the pathway proteins PI3K and AKT, we found that AOF pretreatment could increase the expression of p-PI3K and p-AKT in PC12 cells. On the other hand, the protective effect of AOF was significantly decreased after the use LY294002, the PI3K/AKT signaling pathway inhibitor. These results indicate that AOF can protect cells from apoptosis induced by oxidative stress through the PI3K/AKT signaling pathway (Figure 14). Obviously, we still have some shortcomings in this experiment. For example, when researching the active ingredients in AOF, we only used water extracts for verification, no ethanol or other extracts. In addition, the experimental verification part has only been verified *in vitro*, and no relevant textual research has been carried out on the *in vivo* pharmacodynamics. But in the future, the above shortcomings will also be the direction and goal of our continued research.

## Conclusion

This study combines network analysis and *in vitro* experiment to explore the main active components and related molecular mechanisms of AOF against AD. In the experiment, 39 compounds were analyzed and obtained. After screening their potential targets, they were intersected with the obtained AD therapeutic targets. After GO and KEGG analysis, it was concluded that the anti-AD effect of AOF may be related to apoptosis caused by oxidative stress, and the main pathway is PI3K/AKT signaling pathway. Finally, related experiments confirmed that AOF can inhibit the apoptosis induced by oxidative stress through the PI3K/AKT signaling pathway, thereby exerting an anti-AD effect.

## Data Availability

The original contributions presented in the study are included in the article/supplementary material further inquiries can be directed to the corresponding authors.

## Abbreviations

AD, Alzheimer's disease; AOF, Alpiniae oxyphyllae Fructus; SOD, Superoxide dismutase; CAT, Catalase; GSH-Px, Glutathione peroxidase; MMP, Mitochondrial membrane potential; FDA, Food and Drug Administration; Aβ, Amyloid beta; FBS, Fetal bovine serum; PBS, Phosphate buffer saline; DMSO, Dimethyl sulfoxide; CCK-8, Cell counting kit-8; OMIM, Online Mendelian Inheritance in Man database; DMTD, Drug-Molecular-Target-Disease Network; KEGG, Kyoto Encyclopedia of genes and genomes; AO/EB, Acridine orange/ethidium bromide; PVDF, Polyvinylidene fluoride; SD, Standard deviations; P, Biological processes; CCs, Cellular components; MFs, Molecular functions
